# Pre-hospital pulse glucocorticoid therapy in patients with ST-segment elevation myocardial infarction transferred for primary percutaneous coronary intervention: a randomized controlled trial (PULSE-MI)

**DOI:** 10.1186/s13063-023-07830-y

**Published:** 2023-12-15

**Authors:** Jasmine Melissa Madsen, Laust Emil Roelsgaard Obling, Laura Rytoft, Fredrik Folke, Christian Hassager, Lars Bredevang Andersen, Niels Vejlstrup, Lia Evi Bang, Thomas Engstrøm, Jacob Thomsen Lønborg

**Affiliations:** 1 Department of Cardiology, The Heart Center, Rigshospitalet, Copenhagen University Hospital, Copenhagen, Denmark; 2 Copenhagen Emergency Medical Services, Copenhagen, Denmark, and Department of Cardiology, Copenhagen University Hospital Herlev, Copenhagen, Denmark; 3 Region Zealand Emergency Medical Services, Naestved, Denmark

**Keywords:** ST-segment elevation myocardial infarction, Pre-hospital intervention, Randomized controlled trials, Inflammation, Reperfusion Injury, Steroid, Cardioprotection, Cardiac magnetic resonance

## Abstract

**Background:**

Inflammation in ST-segment elevation myocardial infarction (STEMI) is an important contributor to both acute myocardial ischemia and reperfusion injury after primary percutaneous coronary intervention (PCI). Methylprednisolone is a glucocorticoid with potent anti-inflammatory properties with an acute effect and is used as an effective and safe treatment of a wide range of acute diseases. The trial aims to investigate the cardioprotective effects of pulse-dose methylprednisolone administered in the pre-hospital setting in patients with STEMI transferred for primary PCI.

**Methods:**

This trial is a randomized, blinded, placebo-controlled prospective clinical phase II trial. Inclusion will continue until 378 patients with STEMI have been evaluated for the primary endpoint. Patients will be randomized 1:1 to a bolus of 250 mg methylprednisolone intravenous or matching placebo over a period of 5 min in the pre-hospital setting. All patients with STEMI transferred for primary PCI at Rigshospitalet, Copenhagen University Hospital, Denmark, will be screened for eligibility. The main eligibility criteria are age ≥ 18 years, acute onset of chest pain with < 12 h duration, STEMI on electrocardiogram, no known allergy to glucocorticoids or no previous coronary artery bypass grafting, previous acute myocardial infarction in assumed culprit, or a history with previous maniac/psychotic episodes. Primary outcome is final infarct size measured by late gadolinium enhancement on cardiac magnetic resonance (CMR) 3 months after STEMI. Secondary outcomes comprise key CMR efficacy parameters, clinical endpoints at 3 months, the peak of cardiac biomarkers, and safety.

**Discussion:**

We hypothesize that pulse-dose methylprednisolone administrated in the pre-hospital setting decreases inflammation and thus reduces final infarct size in patients with STEMI treated with primary PCI.

**Trial registration:**

EU-CT number: 2022–500762-10–00; Submitted May 5, 2022. ClinicalTrials.gov Identifier: NCT05462730; Submitted July 7, 2022, first posted July 18, 2022.

## Administrative information

Note: the numbers in curly brackets in this protocol refer to SPIRIT checklist item numbers. The order of the items has been modified to group similar items (see http://www.equator-network.org/reporting-guidelines/spirit-2013-statement-defining-standard-protocol-items-for-clinical-trials/).

**Table:** 

Title {1}	Pre-hospital Pulse Glucocorticoid Therapy in Patients with ST-Segment Elevation Myocardial Infarction Transferred for Primary Percutaneous Coronary Intervention: A Randomized Clinical Trial (PULSE-MI)
Trial registration {2a and 2b}.	EU-CT number: 2022–500762-10–00; ClinicalTrials.gov Identifier: NCT05462730
Protocol version {3}	Version: 4.0 of March 8, 2023
Funding {4}	The study is supported by the Clinical Research Unit, Department of Cardiology, Rigshospitalet, Copenhagen University Hospital, Denmark. JMMs and LOs salary is supported by the Rigshospitalet Research Foundation (E-22670–08) and (E-22652–04). FF has received research grant from Novo Nordisk Foundation (NNF19OC0055142). LRs salary is supported by Novo Nordisk Foundation (NNF18OC034384).
Author details {5a}	JMM, LO, LR, CH, NV, LB, TE, JL: Department of Cardiology, The Heart Center, Rigshospitalet, Copenhagen University Hospital, Copenhagen, Denmark FF: Copenhagen Emergency Medical Services, University of Copenhagen, Copenhagen, Denmark, and Department of Cardiology, Copenhagen University Hospital Herlev, Copenhagen Denmark LA: Region Zealand Emergency Medical Services, Naestved, Denmark
Name and contact information for the trial sponsor {5b}	Thomas Engstrøm, Professor, MD, PhD, DMSc, Dept. of Cardiology, The Heart Center, Rigshospitalet, Copenhagen University Hospital, Copenhagen, Denmark Email: Thomas.engstroem@regionh.dk
Role of sponsor {5c}	The sponsor maintains authority over all aspects of the trial including design, management, interpretation of results and publication.

## Introduction

### Background and rationale {6a}

In patients with ST-segment elevation myocardial infarction (STEMI), early reperfusion is pivotal to shorten time of ischemia and thereby reduce myocardial damage (infarct size) [1, 2]. Primary percutaneous coronary intervention (PCI) including stent implantation is the preferred revascularization strategy in these patients in order to improve prognosis [3, 4]. One-year mortality following STEMI has decreased to around 10%, yet a substantial number of patients with STEMI still develop clinical heart failure (HF) (22%) [1, 5–7]. Infarct size is a main driver of mortality and heart failure following STEMI and means to reduce infarct size are widely requested [7]. Although restoration of blood flow reduces infarct size, a phenomenon known as “*reperfusion injury*” may prevent myocardial salvage and in some patients be responsible for up to 50% of the final myocardial damage [8]. Reperfusion injury occurs immediately after the restoration of blood flow, and thus early treatment is probably necessary to prevent reperfusion injury. As 60% of patients with STEMI have achieved reperfusion on initial coronary angiogram (CAG), very early treatment immediately after diagnosis of STEMI on the electrocardiogram (ECG) may be necessary to prevent reperfusion injury [6, 7, 9]. Very early treatment may also have an impact on the injury caused by ischemia [5].

Inflammation has been suggested as an important mediator for ischemia–reperfusion injury, and inflammation is induced immediately after the onset of acute myocardial ischemia and is exacerbated following reperfusion [7, 10]. Thus, inflammation per se may drive excessive cardiomyocyte death resulting in decreased contractility, remodeling of the ventricle, and increased infarct size, which are components that may contribute to a reduced left ventricular (LV) ejection fraction (EF) and subsequently HF and death. Hence, preventing or reducing inflammation is a key target for improving prognosis in patients with STEMI.

Glucocorticoid, either endogenous or via administration, is a main driver in the regulation of the systemic inflammatory response [11, 12]. Glucocorticoid mediates two different physiological responses: a slow working genomic effect mediated by glucocorticoid receptor occupation, gene transcription, and translation within the cells which is induced within hours, and a non-genomic effect, which is induced rapidly (< 15 min) after administration via plasma membrane-bound receptors, independent of cytosolic receptor stimulation [13, 14]. Therefore, the non-genomic actions may secure that the anti-inflammatory effect is initiated quickly to protect the myocardium immediately, while the genomic actions potentially protect the tissue in the subsequent period. In the 1970s and 1980s, glucocorticoids in relation to STEMI were studied in animal studies and showed promising results, yet clinical studies showed conflicting results [12, 15, 16]. These studies were, however, performed prior to the reperfusion era and in-hospital [15]. Treatment with glucocorticoid in the pre-hospital setting, close to symptom debut, may therefore reduce the myocardial injury following a reperfused STEMI. Taken all together, administration of 250 mg methylprednisolone in the pre-hospital setting may offer advantages in patients with STEMI. The expected effect may translate into reduced final infarct size, better clinical outcomes including fewer patients with HF, and subsequently reduced mortality.

## Objectives {7}

### Primary objective

The primary objective is to determine whether 250 mg methylprednisolone in patients with STEMI administrated in the pre-hospital setting reduces final infarct size evaluated by 3 months cardiac magnetic resonance (CMR).

### Secondary objective

The secondary objective is to determine the effects of methylprednisolone on key CMR efficacy parameters, clinical endpoints in terms of mortality and hospitalization for HF at 3 months, peak of cardiac markers, and safety. All objectives are specified in the “Outcomes {12}” section.

### Hypothesis

In patients with STEMI referred for primary PCI, 250 mg methylprednisolone administered in the pre-hospital setting limits reperfusion injury and reduces final infarct size measured by late gadolinium enhancement (LGE) on CMR at 3 months after a STEMI.

## Methods: participants, interventions, and outcomes

### Study setting {9}

The study will be carried out throughout the Emergency Medical Services in Region Zealand and Capital Region of Denmark (approximately 2.7 million citizens) and chaired at the Department of Cardiology, Rigshospitalet, Denmark.

### Eligibility criteria {10}

Patients with STEMI will be screened and consecutively included in the ambulance prior to acute CAG at Rigshospitalet, Denmark. Eligibility will be checked by the referring on-call cardiology fellow (RD) at Rigshospitalet. If the patient is eligible, the including RD will ask the ambulance staff by telephone to include and randomize the patient, and thus the study medicine will be administered immediately hereafter in the ambulance.

Inclusion criteria

1. Age ≥ 18 years
2. Acute onset of chest pain with < 12 h duration
3. STEMI as characterized on ECG by one of the following:
  1. At least two contiguous leads with ST-segment elevation ≥ 2.5 mm in men < 40 years, ≥ 2 mm in men ≥ 40 years, or ≥ 1.5 mm in women in leads V2–V3 and/or ≥ 1 mm in the other leads,
  2. Presumed new left bundle branch block with ≥ 1 mm concordant ST-segment elevation in leads with a positive QRS complex, or concordant ST-segment depression ≥ 1 mm in V1–V3, or discordant ST-segment elevation ≥ 5 mm in leads with a negative QRS complex,
  3. Isolated ST depression ≥ 0.5 mm in leads V1–V3 and ST-segment elevation (≥ 0.5 mm) in posterior chest wall leads V7–V9 indicating posterior acute myocardial infarction (AMI),
  4. ST-segment depression ≥ 1 mm in eight or more surface leads, coupled with ST-segment elevation in aVR and/or V1 suggesting left main—or left main equivalent—coronary obstruction.

Exclusion criteria

1. Initial presentation with cardiac arrest (out of hospital cardiac arrest (OHCA))
2. Known allergy to glucocorticoid or known mental illness with maniac or psychotic episodes
3. Patients with previous AMI in the assumed culprit artery
4. Previous coronary artery bypass graft (CABG)
5. Unable to read and understand Danish
6. Other subtypes of AMI than type 1 [17].

A differentiation betweena classic type 1 AMI with plaque rupture and thrombosis formation and a type 2 AMI with other reasons for ST-segment elevation (e.g., takotsubo, myocarditis, pericarditis, spontaneous coronary artery dissection, coronary embolism, coronary spasm, anemia, etc [17].) can only be made after further evaluation including initial CAG and thus after inclusion. Therefore, patients that meet this exclusion criteria may subsequently be excluded. Any patient with a type 2 AMI or who does not fulfill any other inclusion or exclusion criteria (post-randomization exclusions) will be excluded from the main analyses but followed for safety and potential post hoc analysis.

### Who will take informed consent? {26a}

Due to the acute nature of STEMI and the temporarily inability of the patient to provide informed consent in the pre-hospital setting, enrollment will occur as soon as possible and prior to obtaining informed consent. The patient will provide informed consent as soon as possible following the acute initial treatment (including primary PCI), once the patient is clinically stable. The informed consent form (ICF) will be filled out and signed by the patient in the encrypted database program REDCap. In patients with STEMI unable to provide informed consent following primary PCI, consent will be obtained by an independent legally designated representative who will sign the ICF. The legally designated representative consent will be followed by informed consent and signature of the ICF by the patient, once the patient is stable and able to provide consent. If the patient remains unable to provide informed consent three days following admission, the closest relative to the patient and legally designated representative will provide consent on behalf of the patient and sign the ICF as soon as possible. The patient will subsequently provide informed consent as soon as possible. Approval from the authorities can be seen in the “Ethics approval and consent to participate {24}” section.

### Additional consent provisions for collection and use of participant data and biological specimens {26b}

A research biobank is set up with blood samples taken at admission, following approximately 24 h and at 3 months. Any future analyses of unused research biobank material associated to this project will require ethics committee approval and additional consent.

## Interventions

### Explanation for the choice of comparators {6b}

We will investigate the well-known, widely used, and highly potent glucocorticoid, methylprednisolone, administrated as single pulse-dose therapy in the pre-hospital setting in patients with STEMI. Pulse glucocorticoid therapy was first used for acute rejection following kidney transplantation [18] and is defined as treatment with $\ge$ 250 mg prednisolone or equivalent for one or more days [19]. Pulse glucocorticoid therapy is now used as an effective and safe treatment of a wide range of diseases such as rheumatic diseases, dermatologic diseases, optic neuritis, multiple sclerosis, and glomerulonephritis [20]. The beneficial acute effects of pulse glucocorticoid therapy in these conditions are thought to be mediated by the non-genomic effects of glucocorticoids via plasma membrane-bound receptors, and the estimated complete glucocorticoid receptor occupation is reached at approximately 100 mg methylprednisolone, reaching maximum activation around 250 mg [20, 21]. Genomic mechanisms cannot explain the beneficial effects of glucocorticoid doses > 100 mg, and the extent of receptor activation is directly dependent on methylprednisolone dosage [22]. Methylprednisolone is easy to administrate and has an acute effect, and implementation of methylprednisolone administration in the pre-hospital setting is feasible to be a part of the routine clinical care of patients with STEMI. Placebo is chosen as a comparator. The study medicine is approved and recommended for other medical conditions and is expected to be of minimal risk to patients.

### Intervention description {11a}

The study medicine is 2 × 125 mg/2 mL methylprednisolone, a total of 250 mg/4 mL, which comes as a sterile powder with preservative-free isotonic NaCl as diluent. The study medicine takes *30 to 60 s* to mix and needs to be used within 48 h when opened. The placebo will be 0.9% NaCl in 4 mL ampoules. The infusion of both methylprednisolone and placebo will be done over a period of *5 min* in the pre-hospital setting. The Danish “Summary of Product Characteristics” (SPC) for Solu-Medrol (methylprednisolone) recommends infusion of 250 mg methylprednisolone over a period of $\ge$ 5 min [23]. This is due to the potential side-effects of a rapid infusion of methylprednisolone. These potential side-effects are, however, not well supported by the literature.

### Criteria for discontinuing or modifying allocated interventions {11b}

A patient can withdraw from the study at any time, or when medically necessary, as judged by the investigator. If withdrawal of consent occurs, the data of the patient will be used until the date of withdrawal. If the patient withdraws consent due to an adverse event (AE) or adverse reaction (AR), the patient will be followed until the AE/AR is resolved.

The dose of the study medication will be fixed. In case of any clinical signs of an allergic reaction or severe side effects are suspected, the infusion will be terminated immediately. No dosage adjustments are allowed.

### Strategies to improve adherence to interventions {11c}

All including RD’s at Rigshospitalet, legally designated representative, and ambulance staff have relevant knowledge of the study from the handing out of the study protocol, protocol summaries, teaching seminars, instructive video, and pocket cards as well as via e-mail and on-going project recaps. None of the including RDs, legally designated representative, or ambulance staff are involved with the trial except for screening, enrollment, and inclusion of the patients.

The including RD has all necessary information about the patient through medical records, and screening of the patients does not delay the treatment or intervention. The study medicine is easy to administrate and is of low risk of interfering with or delaying any other treatment.

All personnel at Rigshospitalet, Denmark (e.g., nurses, PCI operators), have relevant information about the trial through teaching seminars, instructive video, written information, and/or pocket cards. All parties involved in the trial have been trained specifically in the trial-specific procedures.

### Relevant concomitant care permitted or prohibited during the trial {11d}

All patients included in the trial will be treated according to standard procedures in the pre-hospital setting. Primary PCI and antithrombotic regimens will be performed according to standard procedures and international guidelines. The follow-up medical treatment will accord with international guidelines and local standard operating procedures. The patients included in the trial will have a closer medical control due to participation and detailed follow-up; however, there is no guarantee that the individual participants achieve any benefits from participating in the trial. The trial will not delay or interfere with standard therapeutic or diagnostic procedures. Included patients are allowed to participate in other trials.

### Provisions for post-trial care {30}

The patients are protected under the General Data Protection Regulation (GDPR) including the processing of personal data (data protection law) and the Danish Health Act. All patients are insured by the Patient Compensation Association (the health system responsible for the trial site).

## Outcomes {12}

### Primary outcome

Final infarct size (% of left ventricle (LV) mass) measured by LGE on CMR 3 months following STEMI.

Secondary outcomes.

1. CMR efficacy outcomes:
  - The extent of microvascular obstruction (MVO) on acute and follow-up CMR
  - Presence of intramyocardial hemorrhage (IMH) on acute CMR
  - Acute and persistent MVO on acute and follow-up CMR (yes/no)
  - Area at risk (AAR) on acute CMR (%LV)
  - Acute infarct size (%LV)
  - Myocardial salvage index (MSI) on acute and follow-up CMR (%)
  - LVEF on acute and follow-up CMR (%)
2. All-cause mortality and hospitalization for heart failure at 3 months
3. Peak Troponin-T and creatine kinase (CK)/CKMB during admission
4. Incidence of adverse events the first seven days of the index event

Exploratory outcomes.

1. Pre-PCI and post-PCI thrombolysis in myocardial Infarction (TIMI) flow
2. C-reactive protein (CRP), leucocyte and differential count, thrombocytes, HbA1c, p-glucose, pro-brain natriuretic peptide (BNP), creatine kinase, creatinine, potassium, and sodium at admission, during admission, and after 3 months
3. Arrhythmia (ventricular tachycardia/ventricular fibrillation) leading to cardioversion from inclusion to hospital discharge
4. Killip class at admission
5. Coronary flow reserve (CFR) and index of microvascular resistance (IMR) following primary PCI
6. LVEF on transthoracic echocardiography during admission and at one to 3 months
7. Biomarkers of inflammation at admission, 24 h after admission, and after 3 months
8. LV mass, LV end-diastolic-volume index, LV end-systolic-volume index, and LV remodeling (change in LV-volume index) on CMR at 3 months
9. All-cause mortality and hospitalization for HF after 1 and 10 years

### CMR imaging

The CMR scanner is of 1.5 Tesla field strength by Siemens. Images are acquired by phased-array body surface coil during breath holds and are ECG triggered. All included patients will undergo a CMR examination (acute CMR) within 5 days of STEMI admission at Rigshospitalet, Denmark, and a CMR examination at 3 months ± 2 weeks (follow-up). No CMR will be done in patients with contraindications to CMR including ferrous metal foreign body, severe claustrophobia, or decreased renal function (eGFR < 30 ml/min). The CMR protocol included cine, native T1-mapping, T2^*^ (only acute CMR), LGE imaging, and aortic flow. Cine images are acquired in 2-, 3-, and4-chamber views, short-axis views, and paracoronal view. The intravenous contrast agent, gadobutrol (Gadovist®, Bayer; 1.0 mmol/ml solution for injection), will be used as a single dose. The dose will be given as 0.15 mmol/kg. In cases of eGFR < 60 ml/min/1.73 m^2^, a dose of 0.10 mmol/kg will be given. Short-axis cine images are acquired right after administration of the contrast injection. LGE imaging will be performed 6–10 min following contrast infusion [24]. The inversion time on LGE is meticulously adjusted for optimal nulling of remote healthy myocardium. Images are acquired in the LV short-axis plane from base to apex covering the whole ventricle.

### Image analysis

All quantitative analyses will be performed in circle cardiovascular imaging (CVI42). End-diastolic LV mass (g), LV end-diastolic volume (mL), LV end-systolic volume (mL), LVEF (%), stroke volume (mL), cardiac output (L/min), cardiac index ((L/min)/m^2^), and peak filling rate (ml/s) are calculated by automatic drawing of the endocardial and epicardial borders in the short-axis cine images. Papillary muscles will be excluded from the LV volume. Drawing of the epi- and endocardial borders are done by automatic contour detection by machine learning. If needed, drawings will be corrected accordingly. The cine 3-chamber view is used as reference. T2-weighted imaging have been used for several years to evaluate AAR; however, several studies have shown good agreement between AAR between contrast-enhanced steady-state free precession (CE-SSFP) and T2-weighted imaging [25–27]. Hence, both methods allow for unbiased quantification of AAR [25]. To assess AAR, CE-SSFP is used. AAR is defined as area of higher intensity compared with remote healthy myocardium and is visually/manually defined [25]. Acute and final infarct size is assessed by LGE images by 5 standard deviations (SD) threshold above visually identified remote healthy myocardium [28]. Hyper-intense areas spread in non-culprit area are excluded from infarct size. In cases of several distinct infarct territories, location of edema on the acute CMR is used to identify the culprit territory. If patients only have a follow-up CMR, the CAG will be used to identify the culprit territory. MVO is defined as hypointense areas within the infarct region on LGE images and is measured manually [29]. IMH is identified on T2^*^ images prior to contrast injection and defined as either hypointense regions within the infarcted area with mean T2^*^ value > 2 SD below T2^*^ value of remote healthy myocardium [30] or T2^*^ value within the infarcted area of ≤ 20 ms [31, 32]. Acute and final MSI is calculated as follows: AAR (g) − acute or final infarct size/AAR (g). All analyses will be performed by a reader blinded to all clinical data, and two independent readers will approve all analyses. In case of discordance between readers, the analysis will be discussed with a fourth blinded reader. Interobserver reproducibility for the analyses will be assessed in 25 randomly chosen patients. A variability of ≤ 5% is accepted.

### Participant timeline {13}

**Fig. 1:**
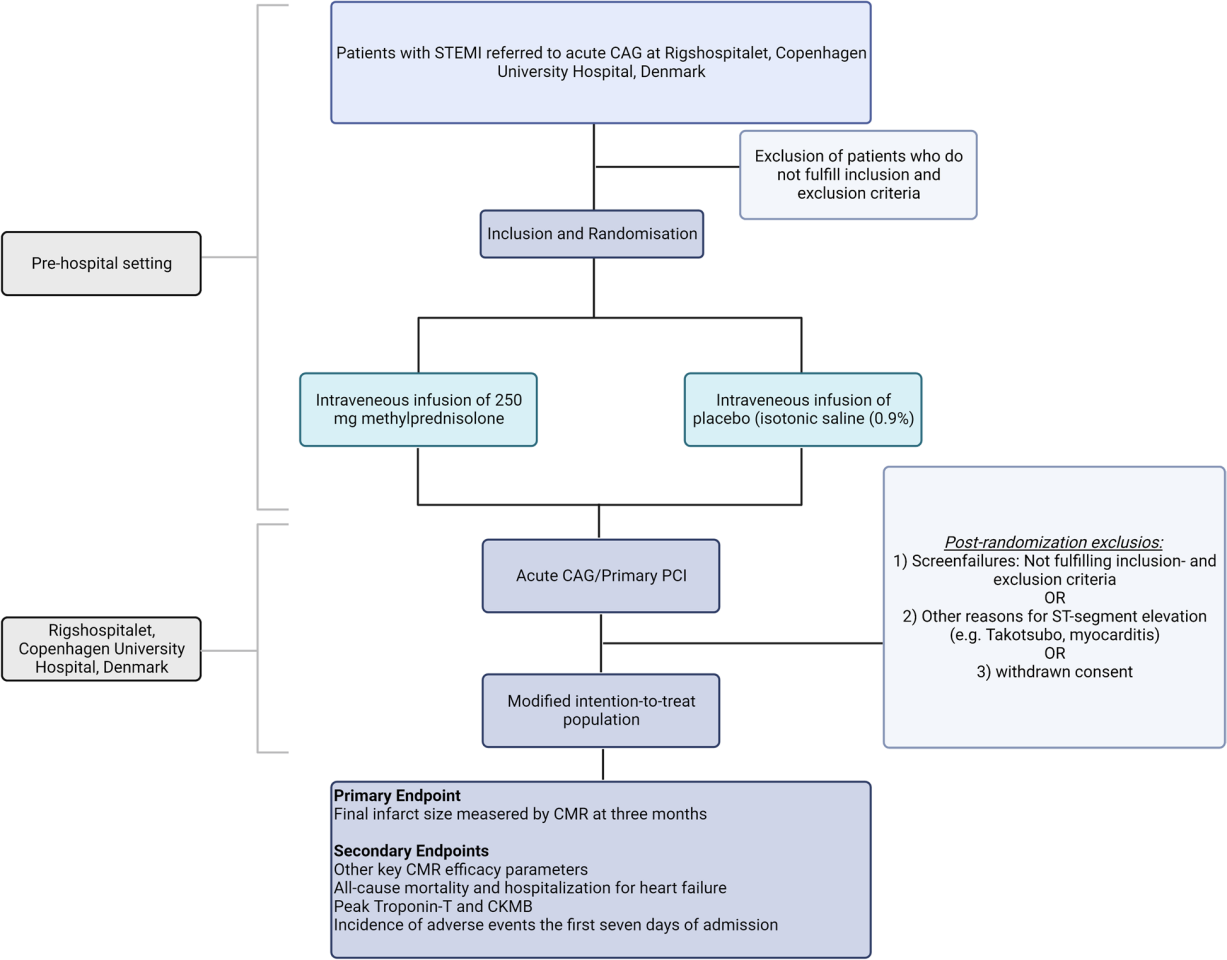
Flowchart of the trial including recruitment, treatment allocation, post-randomization exclusions, and primary and secondary endpoints. Created with BioRender.com. CAG, coronary angiogram; CMR, cardiac magnetic resonance; CKMB, creatine kinase MB; PCI, percutaneous coronary intervention; STEMI, ST-segment elevation myocardial infarction

A flowchart and a schematic diagram of the time schedule are presented in Fig. 1 and Table 1, respectively.

**Table 1:** Time schedule for the trial

	**Study period**					
	**Enrollment**	**Post-allocation**				**Follow-up**
**Timepoint**	**Ambulance**	**During primary PCI**	**Following Primary PCI**	**During admission at Rigshospitalet**		**Days 90**
**Enrolment:**						
**Eligibility screen**	X					
**Informed consent**			X			
**Randomization**	X					
**Interventions:**						
***Infusion of study medicine***	X					
**Assessments:**						
***CMR***				X	X	
***Infarct size***^**a**^				X	X	
***Extent of MVO***^***a***^				X	X	
***IMH***^***a***^				X		
***AAR***^***a***^				X		
***MSI***^***a***^				X	X	
***LVEF***^***a***^				X	X	
***Mortality and hospitalization for heart failure***					X	
***Biochemistry and biobank samples***		X		X	X	
***Coronary physiology***		X				

^a^Evaluated on CMR. *AAR*, area at risk; *CMR*, cardiac magnetic resonance; *IMH*, intramyocardial hemorrhage; *MSI*, myocardial salvage index; *MVO*, microvascular obstruction; *PCI*, percutaneous coronary intervention

### Sample size {14}

The primary outcome is final infarct size (% of LV mass) measured by LGE on CMR at 3 months. Based on results of the CMR sub-studies of the DANAMI-3 trial [33–38], the mean final infarct size measured by LGE on CMR is supposed to be 13% with a SD of 9% in patients with STEMI. To demonstrate a relative reduction in final infarct size of 20% with a two-sided alpha level of 0.05 and a power of 80%, recruitment of 378 patients is needed. A drop-out rate of 30% is expected for the primary outcome but will be adjusted according to the actual drop-out is throughout the trial. However, inclusion will continue until 378 patients have completed the CMR at 3 months.

### Recruitment {15}

Rigshospitalet performs approximately 1000 primary PCI procedures per year [39]. The inclusion is planned to be completed in 12 months, and the 3 months follow-up period will be completed after 15 months. However, the trial will be stopped when 378 patients have completed CMR at 3 months. Following completion of the trial, the hospital records will be accessed at 1 and 10 years to obtain information on mortality and hospitalization for HF.

### Assignment of interventions: allocation

#### Sequence generation {16a}

The pharmacy of the Capital Region of Denmark will pack inbox and label the study medicine and placebo ampoules. The pharmacy is approved by the Danish Health authorities. The randomization will be allocated by the pharmacy and their independent statistician using a random number generator with allocation in a 1:1 fashion with active study medicine and placebo randomized permuted blocks of four. The pharmacy will keep the allocation list and is responsible for generation of the allocation sequence. The pharmacy will not be involved otherwise in the trial. All boxes are identical and has a unique box serial number.

Following preparation, the boxes will be shipped to all the medical emergency unit stations in the Capital Region and Region Zealand of Denmark. All units are manned 24 h 7 days a week. The investigators will make sure that all ambulances always have boxes in storage and ready for use. Before each shift, the ambulance staff will make sure that a minimum of two boxes are stored in each ambulance.

When a patient is screened and included in the trial by telephone by the RD, the ambulance staff will open one of the study medicine boxes and administrate the study medicine (active or placebo). Following the administration of the study medicine, the ambulance staff will fill out a registration form in REDCap. The registration form can be accessed through a QR code on the study medicine box or through an internet browser. For safety, the ambulance staff will also take a photo of the study medicine box, write an inclusion note in the pre-hospital journal, and bring the empty box to the department at Rigshospitalet. To ensure continuous storage at each ambulance, the ambulance staff will get a new study medicine box from the receiving personnel at the hospital department upon arrival. There will be local storages at different hospitals throughout the regions in case the patient is transported to Rigshospitalet by helicopter.

### Concealment mechanism {16b}

Once a patient has been screened and enrolled in the trial, the ambulance staff will fill out the registration form as described above. In the registration form, the paramedic will annotate the following information about the patient: (1) civil registration number, (2) the number of the study medicine box, (3) time of intervention, and (4) if the study medicine was given according to the protocol (yes/no), and if not, why the study medicine was not given. The ambulance staff in the ambulance will sign the registration form following inclusion. Once a registration form has been filled out, the investigators of the trial will be notified by e-mail. The box serial number will also be available in the pre-hospital journal (photo and written note) and/or the delivery of the used study medicine box at the department at Rigshospitalet.

### Implementation {16c}

Allocation of the study medicine will be done by the pharmacy as described in {16a}, and concealment of enrollment and subsequent administration of the study medicine will be done as described in the “Concealment mechanism {16b}” section. The including doctor is the RD at Rigshospitalet who will tell the ambulance staff to include the patient though telephone. The ambulance staff will randomly pick a box and administrate the study medicine within the box in the ambulance. Concealment of trial medicine allocation will follow the procedure described in the “Concealment mechanism {16b}” section. All treating personnel at Rigshospitalet, Denmark, will have no knowledge of the study medicine allocation. Once the trial is completed, the investigators will be informed of the study medicine allocation.

### Assignment of interventions: blinding

#### Who will be blinded {17a}

The ambulance staff is blinded when randomly picking the study medicine box after inclusion in the trial. The treating ambulance staff is unblinded after opening a study medicine box, yet the ambulance staff will not take any further part in the trial, nor the treatment of the patient once arrived at Rigshospitalet. All treating personnel at Rigshospitalet are blinded to the study medicine allocation. Infusion of the study medicine is completed before arriving at Rigshospitalet, and no information about the study medicine allocation will be given to the receiving hospital personnel. Therefore, all other personnel than the ambulance staff are blinded to the treatment allocation. The study medicine allocation will not be unblinded until completion of the entire trial.

#### Procedure for unblinding if needed {17b}

An allocation list is kept in a locked safety cabinet at Rigshospitalet in case of emergency unblinding. Emergency unblinding may occur when ever judged by the investigators. Sponsor and investigators are responsible for the unblinding procedure. Investigators can unblind the study medication allocation without prior contact to the sponsor. According to the ICH-Good Clinical Practice (GCP) guideline 4.3.1, the investigators are responsible for all trial-related medical decisions and can unblind immediately without restrictions.

### Data collection and management

#### Plans for assessment and collection of outcomes {18a}

All patient data will be kept according to the GDPR and the Data Protection Act. Acceptance of the data handling and management will be applied for these institutions before the initiation of the study. The patient data will be kept in an encrypted electronic database, REDCap, and handled as continuous records. The responsibility for the data in the electronic database (eCRF) in REDCap is of the principal investigator. All data will be entered concurrently with the study enrollment by study personnel, and all data will be stored pseudo-anonymously for 25 years. Monitoring of the study will be done by a local GCP unit. The trial will be conducted in compliance with the published trial protocol, the Helsinki Declaration, the GCP guidelines (ICH-GCP), and European and national laws. The first monitoring meeting will be held after inclusion of 2 patients. The following meetings will be held as needed. A full monitoring plan will be elaborated and composed before the initiation of the study by the local GCP unit.

#### Plans to promote participant retention and complete follow-up {18b}

During admission at Rigshospitalet, all eligible and included patients will undergo a CMR examination and will be informed about the 3 months follow-up CMR. The co-primary investigator (JMM) will be responsible for the planning of the follow-up scan. All patients who complete the follow-up scan will also have blood drawn for the research biobank. All patients will be called by telephone at least five times, and patients will be offered transportation renumeration to undertake the CMR scan at 3 months if needed. If the investigator is not able to reach the patient or the patient is unwilling to have the follow-up scan done, the patient will remain in the trial database and followed for all other study parameters.

#### Data management {19}

All data is kept in the database system REDCap. REDCap is hosted and maintained by the IT department of the Capital Region of Denmark.

#### Confidentiality {27}

The patient is protected under the GDPR rules including the processing of personal data (data protection law) and the Danish Health Act, ensuring patient confidentiality throughout the study. All data is kept in an encrypted database, REDCap, and can only the assessed by personnel who have been granted access by the principal investigator. Following completion of the trial, all data including the biobank material analyses will be stored pseudo-anonymously in a secured server.

#### Plans for collection, laboratory evaluation, and storage of biological specimens for genetic or molecular analysis in this trial/future use {33}

Blood samples for the research biobank have a negligible volume for the patient (max. 50 ml throughout the trial). Samples will be drawn during the invasive procedure at admission, 24 h following admission in the ward, and after 3 months before the CMR. All blood samples and data for the research biobank are handled pseudo-anonymously with a unique ID. Following analyses, the research biobank will be terminated in 2024, before 31 December. If any biological material remains following analyses in 2024, the material will be frozen in a biobank for future research and destroyed after 10 years. At this point, no genetic studies are planned, and any further future analyses of unused research biobank material associated with this project will require ethics committee approval.

## Statistical methods

### Statistical methods for primary and secondary outcomes {20a}

The data management workup and statistical analyses will be performed at Rigshospitalet, Denmark. For patients fulfilling all inclusion and exclusion criteria, the treatment groups will be compared as an intention-to-treat principle. Additional as-treated analyses will be performed.

Due to post-randomization exclusions, the primary outcome will be analyzed in a modified intention-to-treat population. The primary outcome will be tested for normality, and *Student’s t-test* or *Mann–Whitney U test* will be used as appropriate to evaluate differences in mean ± SD or median (interquartile range), respectively. Final infarct size will be further evaluated in a linear regression model adjusting for AAR and interaction with treatment allocation evaluated.

In general, differences between group means/medians will be assessed with parametric or non-parametric statistics as appropriate. Chi-square analysis or Fisher’s exact test will be used to test differences between categorical variables. Probability of survival will be displayed using Kaplan–Meier methodology and hazard ratios with 95% confidence interval between groups assessed by Cox proportional hazard models. In case of an event or until the last patient has been followed, the patients will be censored. A two-tailed *P*-value < 0.05 is considered statistically significant.

All statistical analyses will be performed in SAS (version 9.4, SAS Institute, Cary, NC, USA) and R Studio, version 1.2.5001 (RStudio Team (2020). RStudio: Integrated Development for R. RStudio, PBC, Boston, MA; URL: http://www.rstudio.com/).

### Post-randomization exclusions

Patients randomized that subsequently are excluded due to meeting exclusion criteria will be considered post-randomization exclusions. These patients will not be included in the modified intention-to-treat population; however, informed consent from these patients will be obtained, and post hoc analysis in these patients may be done regarding outcome and safety.

### Interim analyses {21b}

No interim analysis will be performed. However, the study will be monitored for safety and efficacy by an independent data and safety monitoring board (DSMB). The role and responsibility of the DSMB are described in the “Composition of the data monitoring committee, its role and reporting structure {21a}” section. The DSMB will provide recommendations about stopping or continuing the clinical trial.

## Methods for additional analyses (e.g., subgroup analyses) {20b}

### Subgroup analyses

The primary outcome will be compared between treatment groups in pre-specified subgroup analyses: age, gender, pre-PCI TIMI flow (0/1 vs 2/3), culprit in left main (LM)/left anterior descending artery (LAD) vs none-LM/LAD, duration of symptom onset to first wire, time from study medicine intervention to first wire, time from first medical contact to first wire, one-vessel disease and multivessel disease, PCI vs CABG, and PCI only. These subgroups will be analyzed using interaction tests. The pre-specified subgroups are factors that are known to influence the clinical course in patients with STEMI.

### Prespecified sub-study

#### Assessment of microcirculation

Prior studies have shown that reperfusion injury following primary PCI in patients with STEMI is equivalent to damage in the microcirculation causing microvascular dysfunction [40]. The microvascular function can be investigated by thermodilution-derived parameters including CFR and IMR measured by a pressure and temperature gauge wire during rest and hyperemia introduced by a 2-min intravenous infusion of adenosine [41, 42]. Prior studies in STEMI patients have shown that IMR has a prognostic value and correlates to microvascular damage resulting in MVO and IMH [43–45]. Moreover, MVO is thought to be the result of reperfusion injury, whereas IMH is a subset of severe microvascular damage due to reperfusion injury [7, 46, 47].

Glucocorticoids may limit reperfusion injury by decreasing the inflammatory response in the microcirculation. To investigate this hypothesis, we will measure CFR and IMR on eligible patients included in the PULSE-MI trial directly after initial primary PCI but before the catheter is removed.

#### Biomarkers

Glucocorticoids is essential in modulating the inflammatory response. In patients with STEMI, several biomarkers such as highly sensitive CRP, pro-BNP, interleukin (IL)-6, IL-1, IL-10, IL-13, IL-18, tumor necrosis factor (TNF)-α, and IL-1β have shown to correlate to prognosis and adverse ventricular remodeling following STEMI [48–50]. However, it is unknown how glucocorticoid may affect these biomarkers in the setting of STEMI. To investigate whether modulating these biomarkers may have a prognostic benefit, blood samples will be drawn at admission in the catheterization laboratory, after 24 h, and at follow-up after 3 months.

### Methods in analysis to handle protocol non-adherence and any statistical methods to handle missing data {20c}

All analyses will be performed in a modified intention-to-treat population. The trial will not be terminated until the completeness of 378 follow-up CMR scans, and therefore, missingness of the primary outcome is not expected.

### Plans to give access to the full protocol, participant-level data, and statistical code {31c}

Data and the protocol will be available upon request and permission of relevant authorities following the completion of the trial.

### Oversight and monitoring

#### Composition of the coordinating center and trial steering committee {5d}

The study will be chaired at Rigshospitalet, Copenhagen University Hospital, Copenhagen, Denmark. The trial steering committee consists of JMM, JL, and TE. The DSMB will be composed as described in the “Composition of the data monitoring committee, its role and reporting structure {21a} section.

#### Daily roles and responsibility of the coordinating center

The trial steering committee is responsible for the daily running of the trial. At least one person of the trial steering committee will be present daily at the coordinating center, Rigshospitalet, to ensure support for running the trial. All participating parties at Rigshospitalet (RD, PCI-operators, nurses, etc.) will have the possibility to contact the steering committee by telephone at all hours. One of the investigators will have daily contact with the RD to ensure support for continuous inclusion in the trial. Furthermore, the steering committee has the contact with the prehospital units in Region Zealand and Capital Region of Denmark. A direct telephone number of one of the investigators is provided for all ambulance staff in the prehospital units to be able to get in touch with the coordinating center. Among themselves, the steering committee will have daily contact and monthly meetings discussing current and possible challenges. The steering committee will monthly send out newsletters to all participating parties in the trial containing information on inclusion status and addressing potential challenges.

#### Composition of the data monitoring committee, its role and reporting structure {21a}

The DSMB will be responsible for safeguarding the interest of trial participants, assessing the safety and efficacy of interventions during the trial and for monitoring the overall conduct of the clinical trial. The DSMB will provide recommendations about stopping or continuing the clinical trial. To contribute to enhancing the integrity of the trial, the DSMB may formulate recommendations relating to the selection, recruitment, and retention of participants, their management, and improving adherence to protocol-specified regiments and procedures for data management and quality control. The key responsibilities of the DSMB are as follows:

1. Reviewing the study protocol
2. Evaluating the quality of ongoing study conduct including accrual rate, adherence to protocol, accuracy, and completeness of data capture
3. Assessing safety and efficacy data by intervention group

The members of this DSMB are professor Lars Køber (chair), professor Nico Pijls, and professor Steen Dalby Kristensen. The DSMB will receive data from an unblinded independent data manager, Emil Fosbøl, who will not be involved in any other aspects of the trial. All meetings will comprise an open meeting (including the steering committee) and a closed meeting (DSMB alone). The DSMB may ask for additional data and events at the discretion of the DSMB.

The DSMB will provide the principal investigator with a written report following every DSMB meeting. The DSMB may ask the steering committee to provide additional data (baseline and outcomes, etc.) before the report is finalized. The DMSB meetings will be held in person or via teleconference.

## Adverse event reporting and harms {22}

### Adverse events/reactions (AE/AR)

AE/ARs that will be recorded daily for the first 7 days after inclusion in the trial. The AE/ARs that will be recorded are infection and any skin complications. All events will be evaluated as “related to the trial medicine/unrelated to the trial medicine” and entered in a pre-specified form in the electronic CRF in REDCap.

### Serious adverse events/reactions (SAE/SAR) and suspected unexpected serious adverse reactions (SUSARs)

The principal investigator and other investigators will record, evaluate, and report all SAEs and SUSARs throughout the trial. Patients will therefore be followed for late-onset SAEs until completion of the trial (1 year). The sponsor of the trial will be registered in the EudraVigilance system prior to initiation of the trial and is responsible for reporting any SUSARs to the EudraVigilance system and the ethics committee as soon as possible, at latest 7 days following awareness of event if life-threatening. If the SUSAR is not life-threating, the event will be reported at latest 15 days following awareness of the event.

### Frequency and plans for auditing trial conduct {23}

The DSMB will meet when the first 25% of the patients have been included and followed until the 3-month follow-up. The DSMB will receive data on the primary outcome. The DSMB will also meet following the inclusion of 50% and 75% of the patients. The DSMB will receive data on the primary outcome. The plan for DSMB meetings in terms of auditing and safety is described in the “Composition of the data monitoring committee, its role and reporting structure {21a}” section. The DSMB will have unlimited access to data upon request.

Monitoring of the study will be done by the GCP unit in Copenhagen. The monitoring will both be on-site and off-site. The first monitoring meeting will be held after the inclusion of 2 patients. The following meetings will be held as needed. The principal investigator will be responsible for all data entered in the eCRF.

### Plans for communicating important protocol amendments to relevant parties (e.g., trial participants, ethical committees) {25}

All amendments have been communicated and authorized by relevant authorities. There will be made no more changes in the current protocol, and the updates have been changed accordingly on the clinical trial registry (https://clinicaltrials.gov/ct2/show/NCT05462730).

### Dissemination plans {31a}

Following completion of the trial, the trial will be unblinded upon acceptance of the steering committee. The results of the trial will be published in an international peer-reviewed journal and presented at national and international congresses. The results will be published regardless of the findings. All authorships will be done according to ICMJE guidelines.

## Discussion

The aim of this trial is to investigate whether administration of a single-pulse-dose glucocorticoid pulse injection in the pre-hospital setting reduces final infarct size and limits reperfusion injury in patients with STEMI. We expect to find a 20% reduction in final infarct size measured by CMR at 3 months following STEMI.

Glucocorticoid has been used for decades, and recent trials have shown that the administration of glucocorticoid in acute diseases such as pneumonia and COVID-19 improves prognosis significantly [51, 52]. Glucocorticoid in the setting of myocardial ischemia has been investigated in experimental, in vivo, and clinical studies in the 1970s and 1980s [12, 15, 16]. Experimental animal studies showed beneficial effects on hemodynamics and infarct size following myocardial ischemia, but clinical in vivo studies have shown conflicting results [12, 15, 16]. Although questions have been raised of impaired infarct heeling in relation to glucocorticoid treatment in the setting of STEMI, the concern is not supported by randomized controlled clinical trials [12, 15, 53, 54]. However, definitive conclusions on glucocorticoids’ efficacy in patients with STEMI are difficult to draw due to different study designs, investigational agents, and medicine dosage [15]. Most importantly, all previous studies in patients with AMI were performed in the era before primary PCI [15] and have focused on treatment after AMI [2]. Furthermore, pulse glucocorticoid therapy administrated intravenously over a short period of time has few side effects compared with long-term oral treatment [55–57]. Based on this knowledge, reperfusion advances, and the role of inflammation in ischemia–reperfusion injury, early treatment with glucocorticoid could have an important therapeutic role in limiting the degree of myocardial injury and improving prognosis in patients with STEMI. The present study is the first to evaluate this effect in a contemporary patient cohort. Contrary to previous studies [15], the present study allows for evaluation of the impact of glucocorticoids on reperfusion injury as intervention is performed pre-hospital and reperfusion is documented by acute CAG upon arrival. Furthermore, the present trial exhibits a pre-hospital setting where the intervention is administrated close to symptom debut, whereas previous trials have performed interventions in-hospital [15].

Inflammation and thus myocardial damage are initiated both by ischemia itself, immediately after occlusion of the artery and subsequently after reperfusion. Up to 60% of patients with STEMI have reperfusion in the pre-hospital setting due to effective antithrombotic treatment with heparin and aspirin, and reperfusion severely adds to the inflammatory burden [9, 10]. Hence, anti-inflammatory intervention in the pre-hospital setting before reperfusion is needed to initiate a potentially rapid non-genomic effect followed by a genomic and prevent reperfusion injury as early as possible. Moreover, as demonstrated in previous studies [5, 35, 58], the potential effect of treating STEMI seems to be more pronounced if the treatment is initiated as early as possible. In addition to reperfusion-induced inflammation, ischemia itself induces inflammation. Hence, initiation of the intervention is needed as close to the debut of symptoms as possible to inhibit the inflammation adequately and effectively in relation to STEMI. By performing intervention in the pre-hospital setting, we expect that the trial will significantly reduce final infarct size translating into fewer patients with heart failure and subsequently reduced mortality following STEMI. Moreover, findings may be more pronoun in patients who generally have larger infarcts and poor prognosis such as anterior STEMI, pre-PCI TIMI-flow 0/1, and longer ischemic time. Hence, reducing infarct size in these specific patients is crucial to improve the clinical course following STEMI and bring down the overall mortality rate. Nevertheless, this trial is proof-of-concept powered to find a reduction in infarct size and not clinical outcomes. In case of positive findings, a larger scale trial will be set up to draw conclusions regarding clinical endpoints.

## Trial status

Protocol: v4.0 of March 3, 2023.

Recruiting status: First patient included in November 2022. Complete recruitment is estimated by the end of September 2023 and completion of follow-up (3 months) CMR scans before the end of January 2024.

## Data Availability

Data required to support the protocol are available upon request and approval from relevant authorities.

## Abbreviations

AAR: Area at risk
AE: Adverse event
AMI: Acute myocardial infarction
AR: Adverse reaction
BNP: Brain natriuretic peptide
CABG: Coronary artery bypass graft
CAG: Coronary angiogram
CFR: Coronary flow reserve
CMR: Cardiac magnetic resonance
CRP: C-reactive protein
ECG: Electrocardiogram
GCP: Good Clinical Practice
GDPR: General Data Protection Regulation
HF: Heart failure
ICF: Informed consent form
IL: Interleukin
IMH: Intramyocardial hemorrhage
IMR: Index of Myocardial Resistance
LAD: Left anterior descending artery
LGE: Late-gadolinium enhancement
LM: Left main
LV: Left ventricle
LVEF: Left ventricular ejection fraction
MSI: Myocardial salvage index
MVO: Microvascular obstruction
OHCA: Out-of- hospital-cardiac arrest
PCI: Percutaneous coronary intervention
RD: Referring on-call cardiology fellow
SAE: Serious adverse events
SAR: Serious adverse reaction
SD: Standard deviation
SPC: Summery for product characteristics
STEMI: ST-segment elevation myocardial infarction
SUSAR: Suspected Unexpected Serious Adverse Reactions
TIMI: Thrombolysis in myocardial infarction
TNF: Tumor necrosis factor
